# Effects of sevoflurane on left ventricular function by speckle-tracking echocardiography in coronary bypass patients: A randomized trial

**DOI:** 10.7555/JBR.37.20230173

**Published:** 2023-12-04

**Authors:** Chanjuan Gong, Xiaokai Zhou, Yin Fang, Yanjuan Zhang, Linjia Zhu, Zhengnian Ding

**Affiliations:** 1 Department of Anesthesiology and Perioperative Medicine, the First Affiliated Hospital of Nanjing Medical University, Nanjing, Jiangsu 210029, China; 2 Department of Cardiology, the First Affiliated Hospital of Nanjing Medical University, Nanjing, Jiangsu 210029, China

**Keywords:** coronary artery bypass grafting, speckle-tracking echocardiography, sevoflurane, transesophageal echocardiography

## Abstract

The present study aimed to dynamically observe the segmental and global myocardial movements of the left ventricle during coronary artery bypass grafting by transesophageal speckle-tracking echocardiography, and to assess the effect of sevoflurane on cardiac function. Sixty-four patients scheduled for the off-pump coronary artery bypass grafting were randomly divided into a sevoflurane-based anesthesia (AS) group and a propofol-based total intravenous anesthesia (AA) group. The AS group demonstrated a higher absolute value of left ventricular global longitudinal strain than that of the AA group at both T_1_ (after harvesting all grafts and before coronary anastomosis) and T_2_ (30 min after completing all coronary anastomoses) (*P* < 0.05). Moreover, strain improvement in the segment with the highest preoperative strain was significantly reduced in the AS group, compared with the AA group at both T_1_ and T_2_ (*P* < 0.01). The flow of the left internal mammary artery-left anterior descending artery graft was superior, and the postoperative concentration of troponin T decreased rapidly in the AS group, compared with the AA group (*P* < 0.05). Compared with total intravenous anesthesia, sevoflurane resulted in a significantly higher global longitudinal strain, stroke volume, and cardiac output. Sevoflurane also led to an amelioration in the condition of the arterial graft. Furthermore, sevoflurane significantly reduced strain improvement in the segmental myocardium with a high preoperative strain value. The findings need to be replicated in larger studies.

## Introduction

Coronary artery bypass grafting (CABG) presents a potent revascularization advantage in patients with multivessel coronary artery disease (CAD)^[1–4]^. However, the complexity of CABG can lead to various perioperative complications, increasing the risk of postoperative readmissions and mortality^[5]^. Optimal intraoperative management can reduce perioperative complications and improve the prognosis of such patients^[6]^.

Sevoflurane is a widely used inhaled anesthetic in clinical applications; however, its use in patients with CAD remains disputable, because recent studies have reported inconsistent outcomes^[7–10]^. Sevoflurane has demonstrated the ability to induce pharmacological preconditioning in cardiac tissues^[11]^, improve postischemic recovery^[12]^, and reduce the use of intraoperative vasopressors^[13]^. However, sevoflurane can also have adverse effects, such as inhibiting cardiac systolic function and causing coronary artery dilation, potentially aggravating myocardial ischemia in the stenosis of the coronary artery inundated area^[14–15]^. Therefore, in patients with CAD, whether sevoflurane should be used and the exact effect of sevoflurane on intraoperative cardiac function remain to be elucidated.

In the last two decades, transesophageal echocardiography (TEE) has become an essential perioperative diagnostic and monitoring instrument for cardiac anesthesia, enabling accurate and quantitative evaluation of cardiac performance in critically ill patients^[16–17]^. Another noteworthy advancement is the development of speckle-tracking echocardiography (STE), which utilizes myocardial strain and strain rate imaging to precisely measure dimensional or deformational alterations^[18–19]^. STE can provide the detailed quantitative and qualitative information on both global and regional cardiac functions, offering intricate myocardial deformation parameters. The evaluation of myocardium strain and strain rate of the left ventricle (LV) has demonstrated their superior diagnostic sensitivity and specificity for certain heart conditions, such as ischemic heart disease and myocarditis^[20–21]^. Because of these technologies, perioperative monitoring of myocardial functions in critically ill patients has gradually changed from a traditional abstraction and generalization to a more visual, quantitative, and precise evaluation.

Few investigators have evaluated the effects of sevoflurane on intraoperative and perioperative LV functions in patients with CAD, using dynamic real-time quantitative analysis. Here, we performed a randomized controlled trial using STE to dynamically observe the segmental and global myocardial movements of the LV during CABG and to appraise the effects of sevoflurane on cardiovascular functions.

## Materials and methods

### Ethical statement

The present prospective and randomized controlled trial was registered in the Chinese Clinical Trial Registry (ChiCTR2000040751). The investigational protocol was approved by the authors' Institutional Review Board (approval No. 2020-SR-079), and each patient participating in the present study provided a written informed consent.

### Patient enrollment

Patients scheduled for an elective off-pump CABG at the First Affiliated Hospital of Nanjing Medical University between May 1, 2020, and April 30, 2021, were recruited. The inclusion criteria were as follows: BMI of 18–28 kg/m^2^; the American Society of Anesthesiologists physical status classification of grade Ⅱ–Ⅲ; heart rate (HR) of 60–100 bpm in sinus rhythm; and no obvious liver or kidney dysfunction. The exclusion criteria were as follows: irregular rhythms, recent myocardial infarction, valvular heart disease, or contraindications to TEE (*e.g.*, esophageal tumor and active upper gastrointestinal bleeding). Patients were sequentially numbered and then randomized into either a sevoflurane-based anesthesia group (the AS group) or a propofol-based total intravenous anesthesia (TIVA) group (the AA group), using a computer-generated random number table. The team members included an anesthetist, two sonographers, and two anesthesia nurses. Preoperatively, Nurse A sealed randomization details, which the anesthetist unveiled on the surgery day. Other team members and patients remained blinded, with the vaporizer and infusion pumps concealed. Sonographer A performed the TEE examination and transferred the dynamic images to the workstation, where they were subsequently analyzed offline by Sonographer B. Nurse B recorded hemodynamic data and collected postoperative data.

### Anesthetic management

All patients received standard monitoring during the surgery, including electrocardiography and invasive blood pressure, oxygen saturation, HR, and bispectral index (BIS, Aspect Medical System, Minneapolis, MN, USA) assessments. General anesthesia was induced with intravenous midazolam (0.05 mg/kg), fentanyl (8 μg/kg), etomidate (0.3 mg/kg), and cisatracurium (0.2 mg/kg) to facilitate tracheal intubation. A pulmonary artery catheter (Edwards Lifesciences, Irvine, CA, USA) was inserted, and three measurement valures of pulmonary capillary wedge pressure (PCWP) were recorded at the end of the trial. The mean of the three PCWP measurement values was applied for the analysis. Subsequently, a TEE probe with a live three-dimensional acquisition capability (X2-7t, Philips, Amsterdam, the Netherlands) was inserted. Another 5 μg/kg of fentanyl was administered before the incision.

In the AS group, anesthesia was maintained with sevoflurane (0.7 to 2.0 minimum alveolar concentration), supplemented by remifentanil and cisatracurium, targeting a BIS of 40–60. In the AA group, the propofol-based TIVA [3–12 mg/(kg∙h)] was used with remifentanil and cisatracurium, aiming for the same BIS range.

### Transesophageal echocardiographic examination

**Figure 1:**
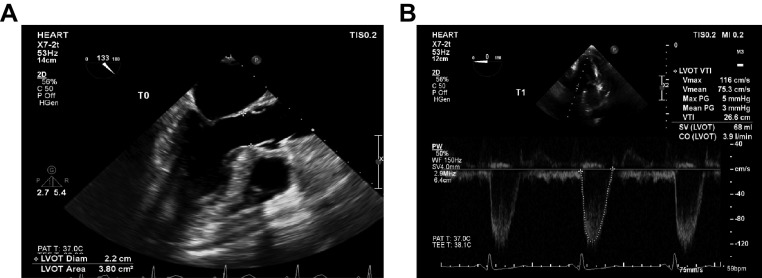
Intraoperative SV and CO measurement.

TEE was performed at three time points: T_0_ (5 min after placement of the TEE probe), T_1_ (after harvesting all grafts and prior to the coronary anastomosis), and T_2_ (30 min after all coronary anastomoses were completed). Standard views, including the four-, two-, and three-chamber views through the middle esophagus (ME4C, 2C, and 3C) with a frame rate of > 50 Hz, a short-axis view through the basal segment of the LV, and pulse Doppler spectrum of the outflow tract through the long-axis view of the LV, were obtained. LV ejection fraction (LVEF) was evaluated by Simpson's biplane technique with standard ME4C and 2C views^[22]^. Stroke volume (SV) was computed from the LV outflow tract velocity using the deep transgastric long-axis view and cross-sectional area of LV outflow utilizing the mid-esophageal long-axis view^[23]^. Cardiac output (CO) was calculated from SV and HR (***Fig. 1***). Dynamic ultrasound images were retained for at least three cardiac cycles, and hemodynamics remained stable during the examinations. Mean arterial pressure (MAP), HR, PCWP, and central venous pressure (CVP) were documented at T_0_, T_1_, and T_2_.

### Speckle-tracking measurement

**Figure 2:**
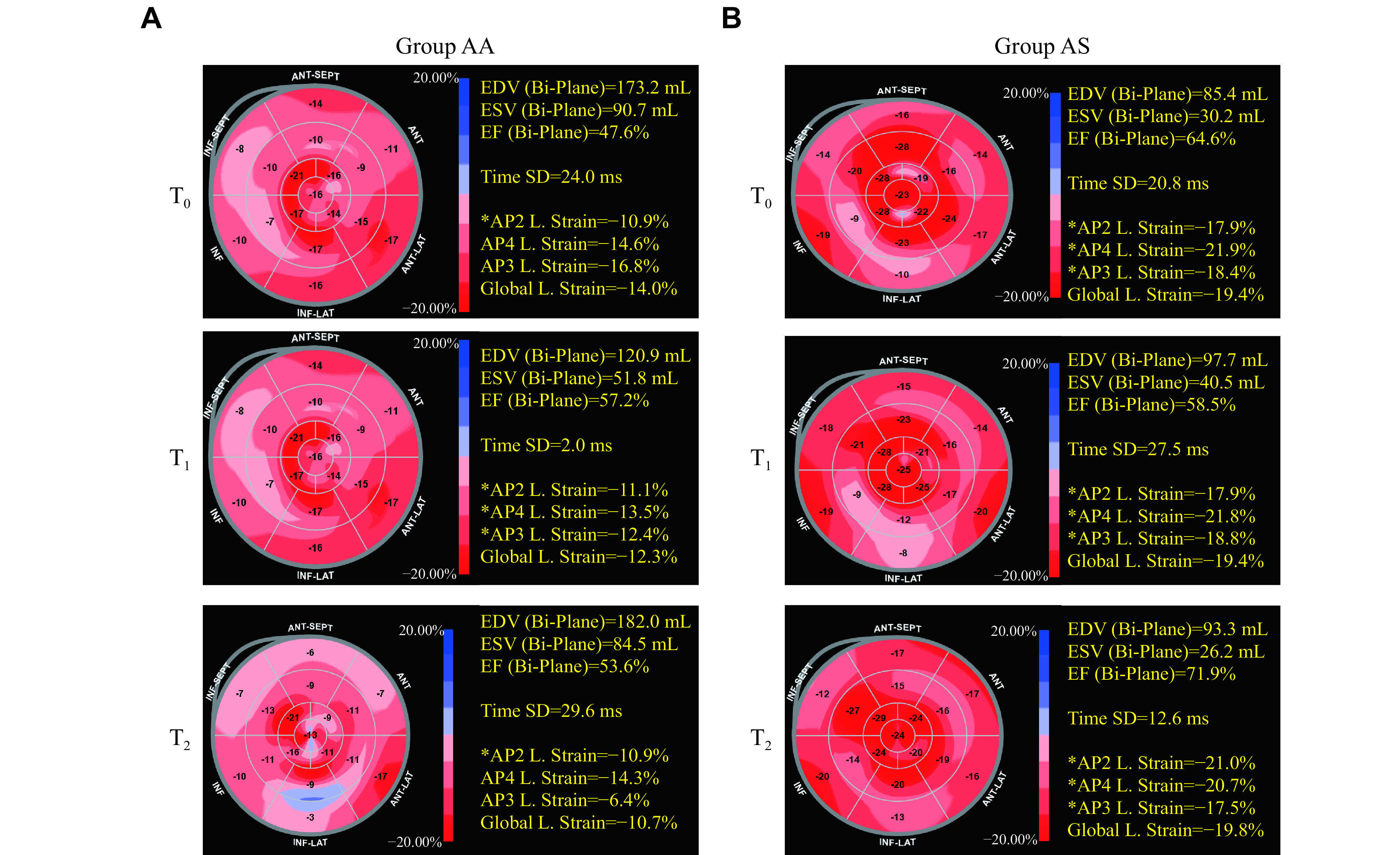
Longitudinal strain bull's eye diagrams.

**Figure 3:**
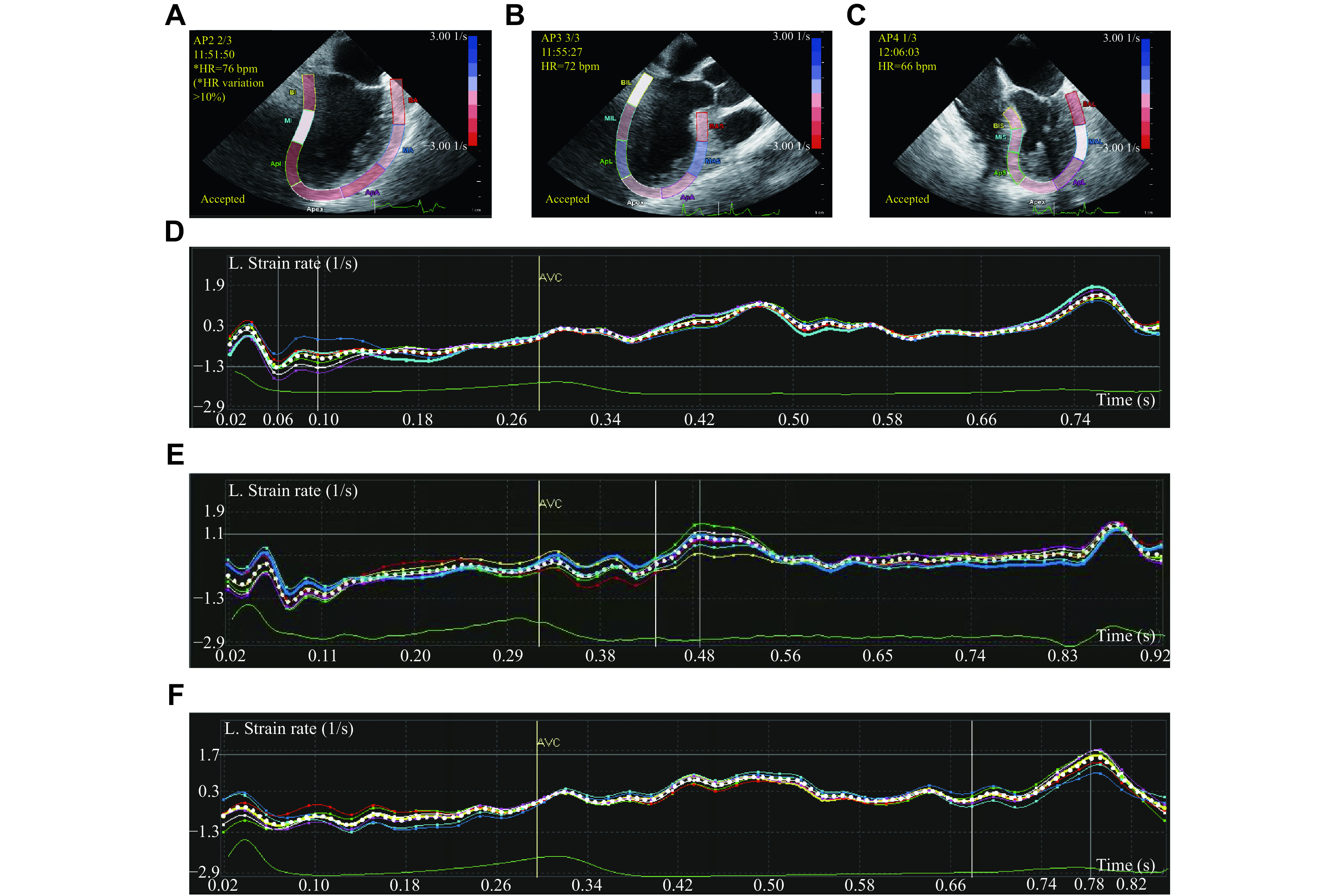
Longitudinal strain rates.

The strain-based indices (SBIs) were measured using a standalone Philips QLab workstation (QLab 10.7, Philips Healthcare, Bothell, WA, USA) with the aCMQ software (Philips Healthcare). The endocardium was traced on an end-systolic frame as the first contour, and the tracking was computed throughout the cardiac cycle. The region of interest (ROI) was defined by approximating the myocardium between the endocardium and epicardium, accounting for wall thickness. The strain value for each myocardial section was computed, and a 17-segment strain bull-eye diagram and global longitudinal strain (GLS) of the LV myocardium were produced (***Fig. 2***). The aortic valve closing (AVC) time was set automatically based on the timing of the minimum volume using the ME3C view. The pinnacle strain rates throughout systole (SRs) were attained from the left of the AVC timeline, while the pinnacle strain rates during early diastole (SRe) and atrial filling (SRa) were obtained on the right of the AVC timeline (***Fig. 3***). The GLS, SRs, SRe, and SRa of the LV were measured in all segments of the three views and averaged to calculate global values at T_0_, T_1_, and T_2_. The segment with the highest longitudinal strain value at T_0_ was defined as the "worst segment". The serial number and longitudinal strain value of the worst segment (WLS) at T_0_, T_1_, and T_2_ and changes in WLS from T_0_ to T_1_ and from T_1_ to T_2_ were recorded.

### Perioperative management and postoperative follow-up

The Ringer's acetate solution and 5% human albumin were used to maintain fluid balance. Dopamine, norepinephrine, metoprolol, and nitroglycerin were administered, if necessary, to preserve stable intraoperative blood flow dynamics, with the pulse rate and arterial pressure within 25% of the baseline measurements. Each patient received heparin (125 U/kg) before anastomosis to preserve an activated coagulation time of > 300 s. After all bridging vessels had been patched, 5 μg/kg of fentanyl and 1 mg/kg of protamine were administered, and the flow and resistance indices of the bridging vessels were recorded. After the surgical procedure, all the subjects were relocated to the intensive care unit. Venous blood was collected from the patients 2 h (D_0_), 24 h (D_1_), and 48 h (D_2_) after surgery to determine the concentration of troponin T (cTnT). The duration of extubation following the operation, the length of hospitalization after the operation, postoperative complications, and other circumstances were documented.

### Outcome measurements

The main outcome criteria for the present study were the GLS of LV at T_0_, T_1_, and T_2_. The second outcome criteria included other SBIs (WLS, SRs, SRe, and SRa) and hemodynamic indices (HR, MAP, CVP, PCWP, SV, CO, and EF) at T_0_, T_1_, and T_2_; intraoperative bloodstream and resistance index (RI) of the arterial graft (left internal mammary artery-left anterior descending coronary branch [LIMA-LAD]); postoperative venous blood cTnT levels at D_0_, D_1_, and D_2_; and postoperative complications during hospitalization.

### Sample size calculation

After conducting a preliminary pilot study involving eight patients in each group, the results indicated that at the T_1_ time point, the mean difference in GLS of the LV between the two groups was 1.72, with a combined standard deviation of 2.01. We adopted a grouped design for quantitative data and opted for a compound symmetric covariance structure for our analyses. We set the test level *α* at 0.05 and the test power 1 − *β* at 0.8 for a two-tailed test. Then, we calculated that a minimum of 23 patients would be required per group. To account for a potential 20% sample attrition rate, we ultimately included 30 patients in each group for the present study.

### Statistical analysis

Data analysis was carried out using SPSS 27.0 (IBM Corp., Armonk, NY, USA). Descriptive statistics, such as demographic and surgical specifics, SBIs, HR, SV, CO, EF, and hemodynamic parameters, were presented as mean ± standard deviation or as median with a 25%–75% interquartile range, depending on whether they were normally distributed. After testing for normality using the Shapiro-Wilk test, Student's *t*-test was employed to analyze normally distributed continuous data, while the Mann-Whitney *U* test was employed for data with non-normal distributions. The Chi-square or Fisher's exact test was implemented to assess categorical data, such as sex and comorbidities. The Wilcoxon signed-rank test was performed for hierarchical data, such as the number of stenotic and bridge vessels. The degree of significance was designated as *P* < 0.05. Graphs were established by the GraphPad Prism 5.0 software (GraphPad, San Diego, CA, USA).

## Results

**Figure 4:**
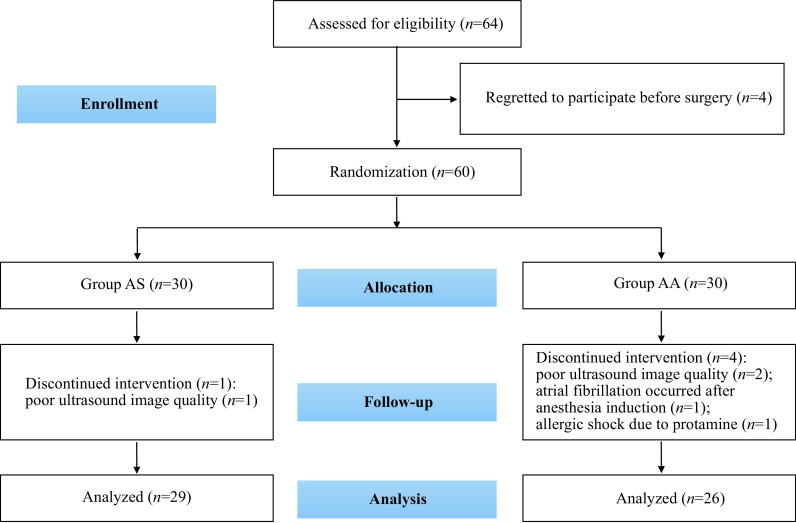
CONSORT diagram for the trial.

Between May 1, 2020 and April 30, 2021, 64 patients who underwent the off-pump CABG and met the inclusion criteria at our institution were eligible. Four patients who declined to participate before the surgery were excluded, and the remaining 60 patients were randomly allocated into two groups. One patient (poor ultrasound image quality, *n* = 1) in the AS group and four patients in the AA group (poor ultrasound image quality, *n* = 2; atrial fibrillation after anesthesia induction, *n* = 1; allergic shock due to protamine, *n* = 1) were not included in the final statistical analysis. The final study included 29 patients in the AS group and 26 patients in the AA group (***Fig. 4***). No significant differences were observed regarding demographic and surgical details between the two groups (***Table 1***).

**Table 1:** Baseline characteristics

Characteristics	Group AS^a^ (*n*=29)	Group AA^a^ (*n*=26)	Statistics^b^	*P*-value
Age (years)	65.8±9.2	67.8±7.8	*t*=0.86	0.40
Sex (male/female)	23/6	19/7	*χ*^*2*^=0.29	0.59
Weight (kg)	71.9±12.3	67.8±12.7	*t*=1.22	0.23
Height (cm)	167.8±7.3	163.8±9.2	*t*=1.8	0.08
Comorbidities [*n* (%)]				
Hypertension	23 (79.3)	22 (84.6)	*χ*^*2*^=0.26	0.61
Diabetes	13 (44.8)	14 (53.8)	*χ*^*2*^=0.46	0.50
Hyperlipidemia	11 (37.9)	10 (38.5)	*χ*^*2*^=0.002	0.97
Smoker	16 (55.2)	12 (46.2)	*χ*^*2*^=0.45	0.50
Vessel stenosis > 75%				
1/2/3	3/7/19	1/7/18	*Z*=0.43	0.67
Combined left main trunk stenosis [*n* (%)]	11 (37.9)	11 (42.3)	*χ*^*2*^=0.11	0.74
Preoperative EF (%)	63.2±2.1	61.9±5.2	*t*=0.45	0.65
^a^Eligible patients were randomized into either the sevoflurane-based anesthesia group (Group AS, *n* = 29) or the propofol-based total intravenous anesthesia group (Group AA, *n* = 26).^b^Data that conformed to a normal distribution were expressed as the mean ± standard deviation (SD) and analyzed using Student's *t*-tests. Data that conformed to a non-normal distribution were expressed as the median (interquartile range) and analyzed using the Mann-Whitney *U* test. Rank data (vessel stenosis) were described as numbers and compared using the Wilcoxon rank-sum test. The Chi-square test was used to compare categorical data (sex, comorbidities, combined left main trunk stenosis). Abbreviation: EF, ejection fraction.				

### Strain-based indices

**Figure 5:**
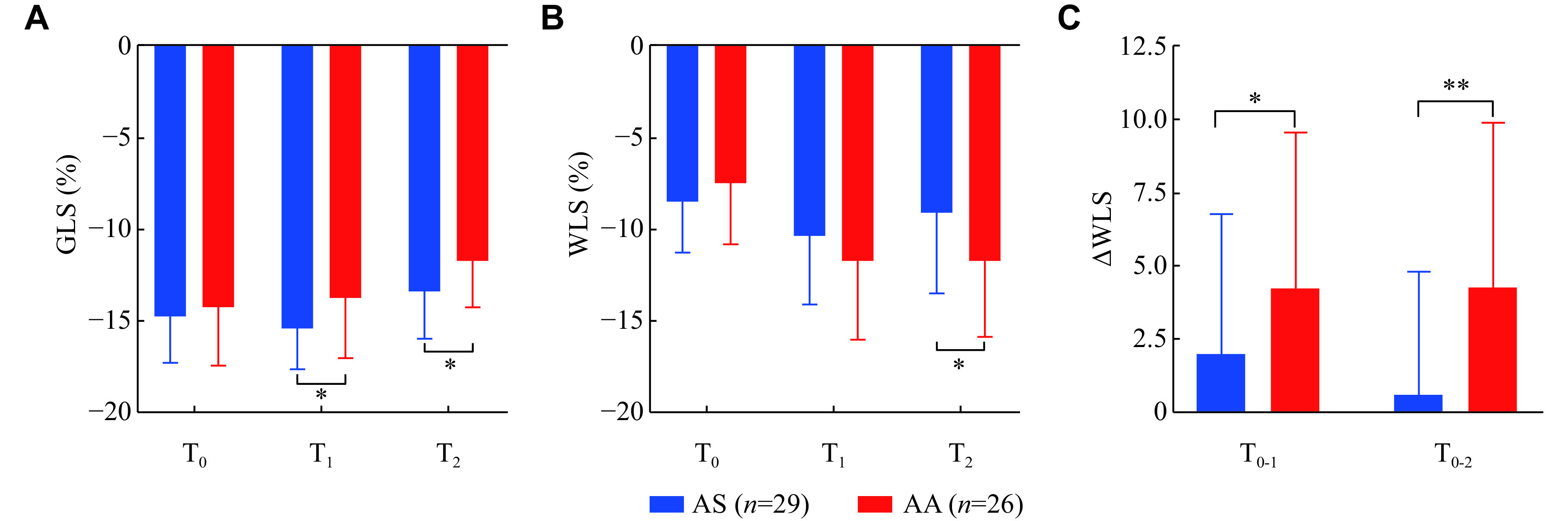
Changes in global and segmental longitudinal strain of two groups.

There was no significant difference in the LV GLS between the AS and AA groups at T_0_, whereas the absolute values of LV GLS at T_1_ and T_2_ were significantly higher in the AS group than in the AA group (T_1_: [–15.5 ± 2.2]% *vs.* [–13.8 ± 3.3]%, *P* = 0.015; T_2_: [–13.4 ± 3.0]% *vs.* [–11.8 ± 2.5]%, *P* = 0.009) (***Fig. 5A***). At T_0_ and T_1_, the WLS did not significantly different between the groups; however, at T_2_, WLS was worse in the AS group than in the AA group ([–9.1 ± 4.4]% *vs.* [–11.8 ± 4.2]%, *P* = 0.025) (***Fig. 5B***). Meanwhile, the improvement in WLS was significantly worse in the AS group than in the AA group at T_1_ and T_2_ (T_0-1_: [1.97 ± 4.80] *vs.* [4.27 ± 5.29], *P* = 0.049; T_0-2_: [0.62 ± 4.20] *vs.* [4.27 ± 5.60], *P* = 0.005) (***Fig. 5C***). The SBIs of SRs, SRe, and SRa, related to diastolic function, showed no significant differences between the groups at any time point (***Table 2***).

**Table 2:** Strain-based indicators at different time points

Variables	T_0_ (mean±SD)					T_1_ (mean±SD)					T_2_ (mean±SD)			
	AS^a^	AA^a^	*t*	*P*		AS^a^	AA^a^	*t*	*P*		AS^a^	AA^a^	*t*	*P*
GLS (%)	−14.8±2.6	−14.3±3.2	0.63	0.53		−15.5±2.2	−13.8±3.3	2.24	0.015^*^		−13.4±3.0	−11.8±2.5	2.43	0.009^**^
WLS (%)	−8.5±2.9	−7.5±3.3	1.18	0.24		−10.4±3.6	−11.8±4.3	−1.25	0.22		−9.1±4.4	−11.8±4.2	−2.31	0.025^* ^
SRs (1/s)	−0.99±0.12	−0.97±0.23	0.64	0.53		−1.03±0.24	−0.98±0.19	0.91	0.37		−1.0±0.23	−1.1±0.22	−0.68	0.50
SRe (1/s)	0.94±0.19	0.96±0.23	0.32	0.75		0.98±0.21	0.97±0.22	−0.19	0.85		0.89±0.17	0.84±0.23	−0.89	0.38
SRa (1/s)	1.02±0.26	0.95±0.28	−0.89	0.38		1.0±0.35	0.94±0.3	−1.02	0.31		1.1±0.25	1.3±0.44	1.59	0.12
^a^Eligible patients were randomized into either the sevoflurane-based anesthesia group (Group AS, *n* = 29) or the propofol-based total intravenous anesthesia group (Group AA, *n* = 26). Data in both groups were normally distributed, expressed as the mean ± standard deviation (SD), and analyzed using Student's *t*-tests. ^*^P < 0.05, ^**^P < 0.01, compared with Group AA. Abbreviations: GLS, global longitudinal strain; WLS, longitudinal strain of segment which had the highest strain value at T_0_; SRa, peak strain rate during atrial filling; SRe, peak strain rate during early diastole; SRs, peak strain rate during systole; T_0_, 5 min after placement of the transesophageal echocardiography probe; T_1_, before coronary revascularization after harvesting grafts; T_2_, 30 min after coronary revascularization.														

### Perioperative hemodynamics

**Figure 6:**
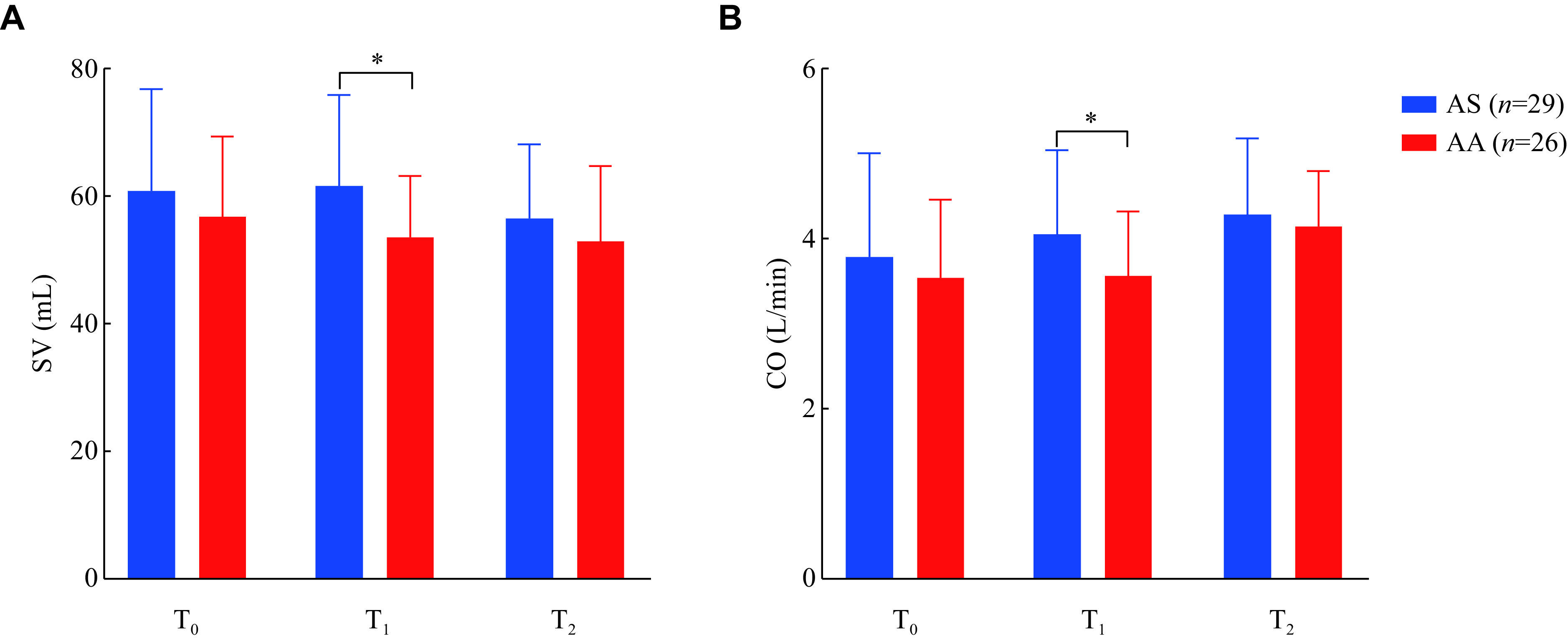
Changes in global and segmental longitudinal strain of two groups.

HR, MAP, CVP, PCWP, and LVEF were not significantly different between the groups at T_0_, T_1_, and T_2_ (***Table 3***). No significant differences were observed in SV and CO between the groups at T_0_ and T_2_, but the SV and CO were higher in the AS group than in the AA group at T_1_ (SV: [61.7 ± 14.3] *vs.* [53.5 ± 9.9], *P* = 0.018; CO: [4.1 ± 1.0] *vs.* [3.6 ± 0.8], *P* = 0.046) (***Fig. 6*** and ***Table 3***).

**Table 3:** Intraoperative hemodynamic variables

Variables	T_0_ (mean±SD)					T_1_ (mean±SD)					T_2_ (mean±SD)			
	AS^a^	AA^a^	*t*	*P*		AS^a^	AA^a^	*t*	*P*		AS^a^	AA^a^	*t*	*P*
HR (bpm)	62.0±9.1	62.9±9.6	0.34	0.74		66.4±10.4	67.3±10.8	0.31	0.76		76.7±9.4	80.4±12.8	1.24	0.22
MAP (mmHg)	78.6±10.0	77.7±11.1	−0.33	0.74		76.2±9.1	77.4±10.2	0.46	0.65		72.1±6.7	69.6±9.7	−1.12	0.27
CVP (mmHg)	6.1±2.5	6.0±2.5	−0.16	0.87		5.9±2.5	6.0±2.3	0.16	0.87		6.2±2.7	5.0±2.7	−1.57	0.12
PCWP (mmHg)	11.1±4.1	9.8±3.6	−1.29	0.20		10.6±4.0	10.5±3.6	−0.09	0.93		10.9±4.2	9.2±3.9	−1.48	0.15
SV (mL)	60.9±16.0	56.7±12.8	−1.07	0.29		61.7±14.3	53.5±9.9	−2.44	0.018^*^		56.6±11.8	52.9±11.9	−1.15	0.26
CO (L)	3.8±1.2	3.5±0.9	−0.83	0.41		4.1±1.0	3.6±0.8	−2.04	0.046^*^		4.3±0.9	4.2±0.7	−0.72	0.48
EF (%)	53.3±6.0	51.1±6.5	−1.27	0.21		52.8±5.1	50.9±8.6	−0.98	0.33		52.5±7.9	52.3±7.8	−0.10	0.92
^a^Eligible patients were randomized into either the sevoflurane-based anesthesia group (Group AS, *n* = 29) or the propofol-based total intravenous anesthesia group (Group AA, *n* = 26). Data in both groups were normally distributed, expressed as the mean ± SD, and analyzed using Student's *t*-tests. ^*^P < 0.05, compared with Group AA. Abbreviations: HR, heart rate; MAP, mean arterial pressure; CVP, central venous pressure; PCWP, pulmonary capillary wedge pressure; SV, stroke volume; CO, cardiac output; EF, ejection fraction; T_0_, 5 min after placement of the transesophageal echocardiography probe; T_1_, before coronary revascularization after harvesting grafts; T_2_, 30 min after coronary revascularization; SD, standard deviation.														

### Intraoperative status of the artery graft

**Figure 7:**
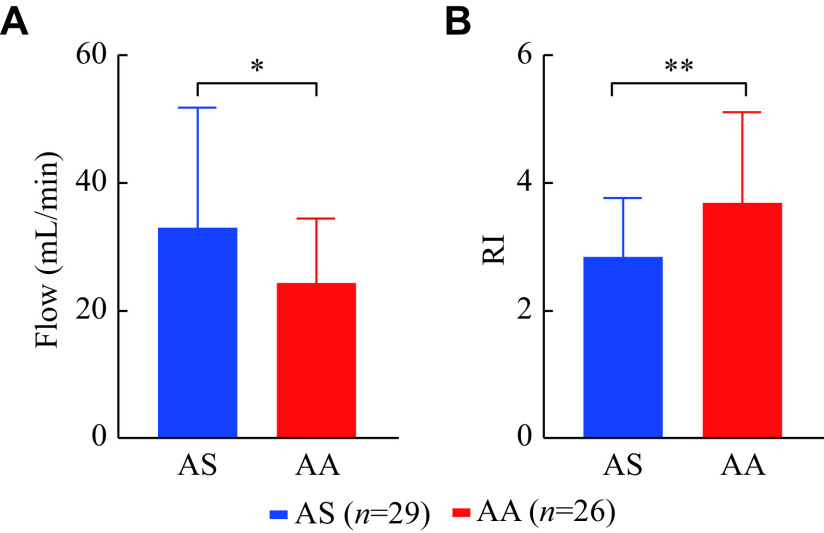
Blood flow and RI of LIMA-LAD graft in two groups.

The blood flow of the LIMA-LAD graft was higher, but the postoperative arterial bridge RI was lower in the AS group than in the AA group (flow: [33.1 ± 18.7] *vs.* [24.4 ± 9.9], *P* = 0.017; RI: [2.84 ± 0.92] *vs.* [3.70 ± 1.40], *P* = 0.006) (***Fig. 7***).

### Postoperative follow-up

The cTnT was higher on D_1_ after surgery in both groups, whereas no significant difference was observed between the groups on D_0_, D_1_, and D_2_. In both groups, cTnT decreased on D_2_, compared with D_1_, and the decrease was greater in the AS group than in the AA group (33.0 [4.3–110.7] *vs.* 12.6 [–10.4–41.6], *P* = 0.048). There were no substantial differences in postoperative complications between the groups during hospitalization (***Table 4***).

**Table 4:** Perioperative details

Variables	Group AS^a^ (*n*=29)	Group AA^a^ (*n*=26)	Statistics^b^	*P*-value
Intraoperative dopamine (mg)	39.3 (34.2–51.9)	45.6 (40.6–55.1)	*Z*=1.73	0.08
Intraoperative norepinephrine (μg)	1467 (1249–1864)	1617 (1496–1988)	*Z*=1.83	0.07
CABG duration (min)	245 (214.5–271.0)	227 (194.8–261)	*Z*=0.93	0.35
Blood loss (mL)	600 (450–900)	550 (500–800)	*Z*=0.30	0.77
Bridge vessels				
1/2/3/4/5	0/4/13/11/1	1/3/9/12/1	*Z*=0.40	0.69
Duration of withdrawing the tracheal tube (h)	24 (20.3–39.5)	23.0 (16.0–31.0)	*Z*=1.22	0.22
Postoperative hospital stay (day)	9.5 (7.0–13.8)	11.0 (5.0–17.0)	*Z*=0.00	1.00
Total incidence of postoperative adverse events				
Infections [*n* (%)]	5 (17.2)	4 (15.4)	*χ*^*2*^=0.19	0.85
Myocardial infarction [*n* (%)]	1 (3.4)	0 (0)	—	1.00
Ventricular fibrillation [*n* (%)]	0 (0)	1 (3.8)	—	0.47
IABP [*n* (%)]	2 (6.9)	2 (7.7)	*χ*^*2*^=0.11	0.91
ECMO [*n* (%)]	0 (0)	1 (3.8)	—	0.47
^a^Eligible patients were randomized into either the sevoflurane-based anesthesia group (Group AS, *n* = 29) or the propofol-based total intravenous anesthesia group (Group AA, *n* = 26).^b^Data following a non-normal distribution were represented as the median (interquartile range) and analyzed with the Mann-Whitney *U* test. Rank data (bridge vessels) were described as numbers and compared using the Wilcoxon rank-sum test. The continuity correction χ2 test (infections, IABP), and Fisher's exact test (myocardial infarction, ventricular fibrillation, and ECMO) were used to compare categorical data. Abbreviations: CABG, coronary artery bypass graft; IABP, intra-aortic balloon pump; ECMO, extracorporeal membrane oxygenation.				

### Discussion

The findings revealed that, in comparison with TIVA, sevoflurane significantly increased LV GLS, SV, and CO, and improved the status of the arterial graft. Furthermore, sevoflurane significantly reduced the strain improvement in the segmental myocardium with a high preoperative strain value. To the best of our knowledge, this is the first study to use transesophageal STE technology to assess the dynamic effect of sevoflurane on cardiac function by observing LV wall movement and intraoperative changes in segmental and global cardiac functions in patients undergoing the off-pump CABG.

STE provides valuable quantitative data about tissue deformation. Studies have demonstrated the role of GLS in identifying subtle myocardial damage and in the specific analysis of endocardial wall deformation properties in patients with CAD^[25]^. Although two studies have reported that STE allows for intraoperative diagnosis (both GLS in on-pump CABG patients and the severity of CAD were correlated with SRE rates in anesthetized patients)^[26–27]^; to our knowledge, no other study has investigated the role of STE in intraoperative anesthesia management to date.

The present study employed the STE technology through TEE to assess intraoperative effects of sevoflurane on cardiac functions. The results indicated that, in comparison with TIVA, sevoflurane enhanced LV GLS before and after coronary revascularization, inconsistent with the results reported by Chennakeshavallu *et al*^[26]^. These differences may be attributed to their choice of on-pump CABG patients, where the cardiopulmonary bypass procedure could mask the effects of sevoflurane. In support of our perspective, Lomivorotov *et al*^[28]^ highlighted the role of cardiopulmonary bypass in postoperative low-cardiac-output syndrome. As the LV myocardium is composed of three layers, coronary artery stenosis first causes subendocardial ischemia that mainly affects longitudinal strain. Thus, the longitudinal strain may be more sensitive in assessing subtle myocardial deformation. The present study also suggests that GLS is a more sensitive indicator than SV and CO for accurately identifying regional myocardial dysfunction.

The present study aimed to evaluate the intraoperative influences of sevoflurane on the LV function. Several cardiac surgery studies used sevoflurane with diverse clinical outcomes^[7–10,29]^. Even if sevoflurane offered cellular protection *via* different mechanisms, such as intracellular signaling pathways^[30]^, mitochondrial function^[31]^, and potassium channels^[32]^, recent studies have suggested that sevoflurane does not significantly reduce mortality or major postoperative complications, such as myocardial infarction, ventilation time and intensive care unit stay^[7–8]^. The myocardial compensatory ability is significantly decreased in patients undergoing CABG, which might explain our finding that sevoflurane improved LV GLS, SV, and CO in patients with CAD, whereas Lindholm *et al*^[33]^ found no significant echocardiographic differences between sevoflurane and TIVA in abdominal aortic surgery.

Some studies indicated that sevoflurane reduced postoperative cTnT and CK-MB levels in critical patients, providing cardioprotection, even with brief pre-ischemia administration^[32,34]^. In the present study, postoperative complications and cTnT discharge did not show notable differences; however, the cTnT levels decreased more quickly on postoperative day 1 and day 2. Consistent with these results, the recent mortality in cardiac surgery (MYRIAD) trial suggested that volatile agents might reduce postoperative myocardial injury in CABG patients^[33]^. The present results also showed that sevoflurane enhanced blood flow but reduced RI in the LIMA-LAD graft. While no direct comparisons exist between sevoflurane and TIVA regarding graft status, sevoflurane is recognized for its significant coronary vasodilatory effects, primarily through potassium channel action, thereby improving myocardial oxygenation^[17,35]^. In contrast, propofol lacks this effect. Kerstern *et al*^[36]^ found that sevoflurane did not cause "coronary steal", but its vasodilation might redirect blood flow from stenotic to normal vessels, leading to a redistribution of myocardial blood flow. In this case, perfusion of the area of coronary artery stenosis that innervates the myocardium may be influenced, and ischemic injury may occur if myocardial oxygen consumption increases. These may elucidate our observation of a reduced strain improvement in high-strain myocardium segments. From these findings, we cannot exclude the sevoflurane's effect of "coronary steal".

The present study has limitations. We did not conduct a long-term follow-up of the patients, as the focus was on the perioperative effects of sevoflurane on cardiac functions. The present cohort comprised patients with CAD, preserved heart function (EF > 50%), and normal LV motion, but future studies should consider patients with milder CAD or heart failure and paradoxical LV motion. Though the sample size was based on the primary endpoint (LV GLS), larger studies targeting other endpoints are warranted.

## Conclusions

The present study demonstrates that GLS is a more sensitive indicator than EF, SV, or CO in evaluating LV systolic function. Meanwhile, compared with TIVA, sevoflurane can increase intraoperative LV GLS, SV, and CO, and improve the status of the arterial graft. The discharge of cTnT reduces at a more accelerated pace on postoperative day 1 and day 2 in patients receiving the off-pump CABG. Furthermore, sevoflurane reduces the strain improvement in the segmental myocardium with a high preoperative strain value. Thus, sevoflurane in patients with severe CAD should be guarded for "coronary steal".

## References

[1] (2021). Coronary artery bypass grafting transit time flow measurement: graft patency and clinical outcomes. Ann Thorac Surg.

[2] (2022). 2021 ACC/AHA/SCAI Guideline for coronary artery revascularization: executive summary: a report of the American College of Cardiology/American Heart Association Joint Committee on Clinical Practice Guidelines. Circulation.

[b3] 3Martuscelli E. Coronary artery bypass graft[M]//Dewey M. Card CT. 2nd ed. Berlin, Heidelberg: Springer, 2014: 191–198.

[4] (2023). A historical literature review of coronary microvascular obstruction and intra-myocardial hemorrhage as functional/structural phenomena. J Biomed Res.

[5] (2022). Cardiorespiratory fitness and the incidence of coronary surgery and postoperative mortality: the HUNT study. Eur J Cardio-Thorac Surg.

[6] (2020). Postoperative complications associated with coronary artery bypass graft surgery and their therapeutic interventions. Future Cardiol.

[7] (2020). Effect of volatile anesthetics on mortality and clinical outcomes in patients undergoing coronary artery bypass grafting: a meta-analysis of randomized clinical trials. Minerva Anestesiol.

[8] (2019). Volatile anesthetics versus total intravenous anesthesia for cardiac surgery. N Engl J Med.

[9] (2019). Inhalation versus intravenous anesthesia for adults undergoing heart valve surgery: a systematic review and meta-analysis. Minerva Anestesiol.

[10] (2020). Volatile anesthetics versus propofol for cardiac surgery with cardiopulmonary bypass: meta-analysis of randomized trials. Anesthesiology.

[11] (2014). Volatile anesthetic-induced preconditioning. Perfusion.

[12] (2021). Sevoflurane preconditioning promotes mesenchymal stem cells to relieve myocardial ischemia/reperfusion injury *via* TRPC6-induced angiogenesis. Stem Cell Res Ther.

[13] (2022). The effects of total intravenous and inhalation anesthesia maintenance on tissue oxygenation in coronary artery bypass graft surgery. Eur Rev Med Pharmacol Sci.

[14] (1991). Direct vasodilation by sevoflurane, isoflurane, and halothane alters coronary flow reserve in the isolated rat heart. Anesthesiology.

[15] (2021). Coronary steal: mechanisms of a misnomer. J Am Heart Assoc.

[16] (2020). Guidelines for the use of transesophageal echocardiography to assist with surgical decision-making in the operating room: a surgery-based approach: from the American society of echocardiography in collaboration with the society of cardiovascular anesthesiologists and the society of thoracic surgeons. J Am Soc Echocardiogr.

[17] (2020). Transesophageal echocardiography in minimally invasive cardiac surgery. Curr Opin Anaesthesiol.

[18] (2017). A test in context: myocardial strain measured by speckle-tracking echocardiography. J Am Coll Cardiol.

[19] (2019). The diagnostic and prognostic value of echocardiographic strain. JAMA Cardiol.

[20] (2019). Echocardiographic assessment of left ventricular systolic function. J Echocardiogr.

[21] (2023). Speckle-tracking echocardiography: state of art and its applications. Minerva Med.

[22] (2015). Recommendations for cardiac chamber quantification by echocardiography in adults: an update from the American Society of Echocardiography and the European Association of Cardiovascular Imaging. J Am Soc Echocardiogr.

[23] (2017). Recommendations on the echocardiographic assessment of aortic valve stenosis: a focused update from the European Association of Cardiovascular Imaging and the American Society of Echocardiography. Eur Heart J Cardiovasc Imaging.

[24] (2022). Effect of volatile anesthesia versus total intravenous anesthesia on postoperative pulmonary complications in patients undergoing cardiac surgery: a randomized clinical trial. J Cardiothorac Vasc Anesth.

[25] (2021). Speckle tracking echocardiography: early predictor of diagnosis and prognosis in coronary artery disease. Biomed Res Int.

[26] (2022). Comparison of effects of sevoflurane versus propofol on left ventricular longitudinal global and regional strain in patients undergoing on-pump coronary artery bypass grafting. Ann Card Anaesth.

[27] (2019). Intraoperative assessment of coronary artery stenosis by 2D speckle-tracking echocardiography: the correlation between peak strain rate during early diastole and the severity of coronary artery stenosis in patients undergoing coronary artery bypass grafting. J Cardiothorac Vasc Anesth.

[28] (2017). Low-cardiac-output syndrome after cardiac surgery. J Cardiothorac Vasc Anesth.

[29] (2022). Effect of volatile anesthetics on myocardial infarction after coronary artery surgery: a post hoc analysis of a randomized trial. J Cardiothorac Vasc Anesth.

[30] (1998). Isoflurane and sevoflurane induce vasodilation of cerebral vessels via ATP-sensitive K^+^ channel activation. Anesthesiology.

[31] (2004). Cardiac preconditioning by volatile anesthetic agents: a defining role for altered mitochondrial bioenergetics. Antioxid Redox Signal.

[32] (2016). Sevoflurane postconditioning affects post-ischaemic myocardial mitochondrial ATP-sensitive potassium channel function and apoptosis in ageing rats. Clin Exp Pharmacol Physiol.

[33] (2014). Analysis of transthoracic echocardiographic data in major vascular surgery from a prospective randomised trial comparing sevoflurane and fentanyl with propofol and remifentanil anaesthesia. Anaesthesia.

[34] (2006). Effects of sevoflurane on cytokine balance in patients undergoing coronary artery bypass graft surgery. J Cardiothorac Vasc Anesth.

[35] (2017). Phosphatidylinositol 3-kinase inhibition induces vasodilator effect of sevoflurane via reduction of Rho kinase activity. Life Sci.

[36] (1994). Perfusion of ischemic myocardium during anesthesia with sevoflurane. Anesthesiology.

